# Control of Persistent *Listeria monocytogenes* in the Meat Industry: From Detection to Prevention

**DOI:** 10.3390/foods14091519

**Published:** 2025-04-26

**Authors:** Belén Romero de Castilla López, Diego Gómez Lozano, Antonio Herrera Marteache, Pilar Conchello Moreno, Carmen Rota García

**Affiliations:** Departamento de Producción Animal y Ciencia de los Alimentos, Facultad de Veterinaria, Instituto Agroalimentario de Aragón -IA2-, Universidad de Zaragoza-CITA, C/Miguel Servet 177, 50013 Zaragoza, Spain

**Keywords:** *Listeria monocytogenes*, prevalence, persistence, meat industry, RTE foods, PFGE, foodborne pathogens

## Abstract

*Listeria monocytogenes* poses a significant food safety risk, particularly in ready-to-eat (RTE) products, due to its persistence in food processing environments. This study aimed to assess the significance of *L. monocytogenes* contamination routes, persistence, and monitoring and control in two Spanish food industries: a fresh pork-cutting industry (Industry A) and an RTE food production industry (Industry B). A total of 698 samples from raw materials, final products, food contact surfaces (FCSs), and non-food contact surfaces (NFCSs) were analyzed using impedanciometry, isolation and identification on chromogenic agars, and molecular typing using serotyping and pulsed-field gel electrophoresis. In Industry A, *L. monocytogenes* contamination increased from 16.7% in raw materials to 53.3% in final products, with four persistent strains detected mainly on FCSs, pointing to their role in pathogen dissemination. In Industry B, the presence of *L. monocytogenes* decreased from 21.2% in raw materials to undetectable levels in the final products. Only one persistent strain was identified, mainly on NFCSs. Serotype 1/2a predominated in both environments. These findings emphasize the importance of robust monitoring, including contamination characterization, for *L. monocytogenes* prevention and control. Strengthening control measures in fresh meat processing and enhancing facility and equipment designs could improve overall hygiene and reduce the persistence of *L. monocytogenes*.

## 1. Introduction

*Listeria monocytogenes* is a significant foodborne pathogen that causes human listeriosis, a severe disease with a high case fatality rate (20–30%) in specific risk groups (neonates, pregnant women, immunocompromised individuals, and the elderly). In 2023, it ranked as the fifth most reported zoonoses in the European Union (EU), with 2952 cases and 19 foodborne outbreaks [1]. The EU reporting rate increased by 5.8% compared to 2022, reaching the highest reported rate and case number since 2007. Listeriosis has the highest hospitalization rate among all zoonoses monitored in the EU, with a fatality rate of 19.7% in 2023 [1].

*L. monocytogenes* is a genetically diverse species classified into four evolutionary lineages (I–IV), five serogroups (IIa, IIb, IIc, IVa, and IVb) and fourteen serotypes (1/2a, 1/2b, 1/2c, 3a, 3b, 3c, 4a, 4ab, 4b, 4c, 4d, 4e, 4h, and 7) [2,3,4,5,6]. Historically, serotype 4b (lineage I, serogroup IVb) has been the most common in human listeriosis cases, despite being less frequently found in food sources. However, over the past decade, serotype 1/2a (lineage II, serogroup IIa) has become dominant in food and environmental samples. It has been increasingly associated with human disease, leading to significant European outbreaks [7,8]. Furthermore, research indicates that serotype 1/2a isolates may have an enhanced ability to form biofilms compared to lineage I, which could explain its persistence in the food industry [9,10,11,12].

Previous risk assessments have identified ready-to-eat (RTE) foods as the primary source of human listeriosis [13,14]. Consequently, Regulation (EC) No. 2073/2005 establishes microbiological criteria for this pathogen in these products and the sampling of processing areas and equipment for RTE foods that may pose an *L. monocytogenes* risk for public health [15]. These regulations are only applicable in RTE-food-producing industries, meaning that the control of this pathogen in these industries is more exhaustive than in those intended for earlier stages in the food chain.

In 2023, the main RTE foods implicated in EU outbreaks were “pig meat and products thereof” and “fish and fish products”. Based on monitoring data, RTE food with meat and meat products ranked second in *L. monocytogenes* prevalence, behind RTE food with fish and fishery products, which had an overall occurrence of 3.1%. The highest prevalence among meat RTE products was observed in bovine meat products (9.9%) and pork products (3.1%) [1].

In Spain, the meat industry is the leading sector within the food and beverage industry, with a turnover of EUR 33,218 million, contributing 2.72% to the Spanish gross domestic product (GDP). Pork production is the main meat activity in this country, representing 4.8% of global production, and ranking as the world’s third-largest producer, behind China (34.3%) and the United States (11.7%) [16].

Food contamination by *L. monocytogenes* is a multifaceted challenge as it is ubiquitously distributed in the environment [7]. This pathogen exhibits adaptive mechanisms enabling survival in adverse conditions, including low temperatures, wide pH ranges (4.6–9.5), high salinity (up to 20%), and limited water availability (a_w_ < 0.90) [17,18]. Its remarkable adaptability and biofilm-forming capacity make controlling this pathogen in processing plants challenging, which facilitates its persistence [8,18,19,20]. Strains with the same PFGE profile isolated repeatedly (at least three times) from the environment or equipment for at least 3 months were considered persistent [21].

Numerous studies have identified the environments of processing plants as the main contamination source, with levels typically increasing along the production line [8,22,23]. Furthermore, several authors state that a typical characteristic of *Listeria* is the persistence of a limited number of genetically similar strains in food processing environments [22,24,25,26,27]. Consequently, specific microbiological control measures must be implemented in processing plants to reduce contamination and enhance cleaning and disinfection (C&D) procedures.

Regulation (EC) No 2073/2005 establishes specific requirements for the control of *L. monocytogenes* in RTE food processing environments. In addition, the new Regulation (EU) 2024/2895, which will enter into force in July 2026, extends the non-detection criteria to 25 g at the end of shelf-life for those RTE foods able to support the growth of *L. monocytogenes* when it has not been demonstrated that the level of *L. monocytogenes* will not exceed the limit of 100 CFU/g throughout the shelf-life of the foods [28]. This new regulatory framework will require operators to reinforce self-monitoring systems, with particular emphasis on the monitoring of environmental contamination in processing plants and on the surveillance of contamination of raw materials.

Given these factors, *L. monocytogenes* represents a significant risk to the food industry, particularly to producers of RTE meat products, being important from both public health and economic perspectives [22]. Even though there is no microbiological criterion for raw meat, as it has rarely been directly linked to outbreaks, several studies emphasize the importance of *L. monocytogenes* contamination in raw meat as a potential source of pathogen entry into the meat processing industry [8,29].

The objective of this study was (i) to identify and assess the significance of *L. monocytogenes* contamination routes, (ii) to evaluate the relevance of persistence in the risk of contamination for its control, and (iii) to assess the importance of monitoring and controlling *Listeria monocytogenes* in an industry dedicated to pork cutting and an industry dedicated to processing RTE foods with pork and beef meat ingredients.

## 2. Materials and Methods

### 2.1. Study Design

Two food industries (A and B) located in Spain were investigated. Industry A is dedicated to fresh pork cutting, and Industry B produces RTE foods with pork and beef meat ingredients. A total of 6 samplings were carried out over 13 months in Industry A (October 2021–November 2022) and 12 samplings over 16 months in Industry B (October 2021–February 2023).

Processing lines and products under study were selected based on (i) contamination risk due to process and product characteristics, (ii) historical microbiological data from each industry, and (iii) expected product use. Industry areas were evaluated according to the risk of activities performed there to identify and prioritize high-risk zones. Surfaces were selected based on (i) food contact, (ii) contact duration, and (iii) ease of surface C&D.

Surfaces were classified as food contact surfaces (FCSs), comprising pre-operational (at least 2 h after C&D) and operational (at the end of processing), and non-food contact surfaces (NFCSs). Raw materials, final products, pre-operational and operational FCSs, and NFCSs were sampled (Supplemental Tables S1 and S2).

**Figure 1:**
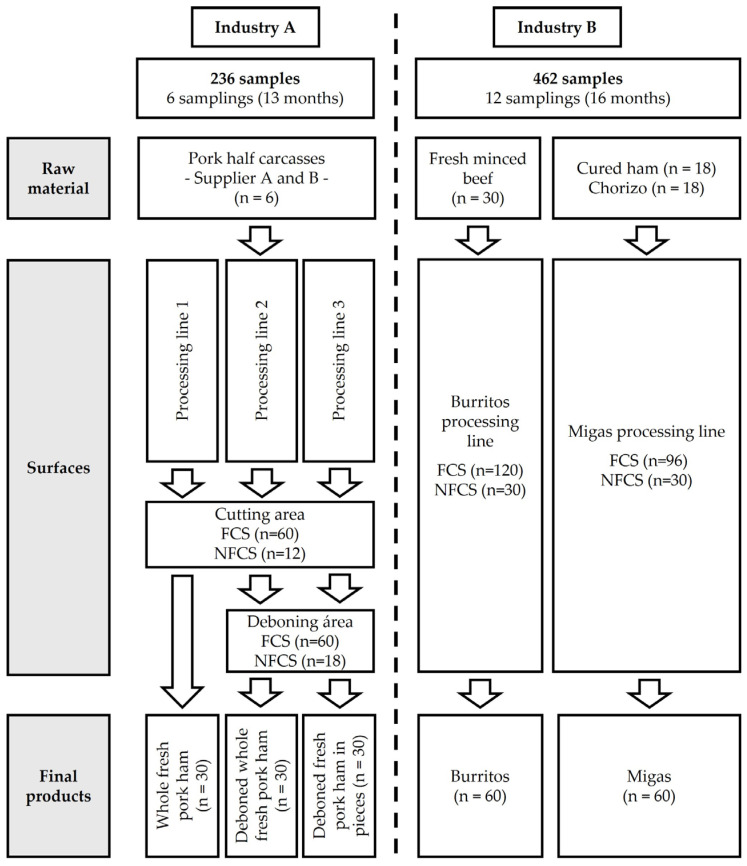
Schematic diagram of the study design for sampling conducted in industries A and B. The figure details the sampling frequency during the study period, as well as the number and type of samples collected (raw materials, surfaces, and final products). The sample categories are abbreviated as “FCS” (food contact surface) and “NFCS” (non-food contact surface).

In Industry A, 236 samples from three fresh pork ham processing lines were analyzed. The common raw material was pork half-carcasses from two different suppliers. The final products analyzed were (i) whole fresh pork ham, (ii) deboned whole fresh pork ham, and (iii) deboned fresh pork ham in pieces. These final products are processed by other industries to produce cured ham (whole fresh pork hams) and cooked ham (deboned whole fresh pork hams and deboned fresh pork hams in pieces). The processing lines are divided into a cutting area common to the 3 processing lines and a deboning area where deboned fresh whole pork ham and deboned fresh pork ham in pieces are processed. In Industry B, a total of 462 samples were analyzed from two RTE food processing lines: a burrito-processing line and a migas-processing line. The raw meat materials analyzed were fresh minced beef (burrito-processing line) and cured ham and fresh chorizo (migas-processing line). The final products were (i) burritos (flour tortillas stuffed with minced beef and vegetables) and (ii) migas (traditional Spanish dish made with fried breadcrumbs mixed with meat products). The sampling areas were divided into a pre-lethal area (area before heat treatment) and a post-lethal area (area after heat treatment) (Figure 1).

### 2.2. Sampling Procedure

Raw materials from Industry A (pork half carcasses) were sampled using moistened VWRC300-0230 (VWR^®^) sampling sponges. Each analytical sample comprised 5 sponges from the internal and external surface rubbings (4 differentiated zones) of 5 half carcasses. Raw materials from Industry B (fresh minced beef, cured ham, and fresh chorizo) were weighed in a homogeneous sample of 25 g [30].

Product samples were collected at the end of processing. In Industry A, pork hams were sampled by the superficial cutting of internal and external zones. For Industry B, burritos were sampled by cutting and weighing the central zone, while migas samples were homogenized before weighing. All product samples adhered to the 25 g sample size specified by ISO 11290-1:2017 [30].

Environmental sampling (FCS and NFCS) was collected using moistened VWRC300-0230 sampling sponges (VWR^®^, Radnor, PA, USA) and sterile swabs for hard-to-reach areas. Sampling was performed using a zigzag movement in both horizontal and vertical directions over a wide unbounded surface, applying constant pressure. Two sponges were used for the operating FCSs to ensure that organic debris did not interfere with the correct analysis of the surfaces [31,32].

All samples were transported refrigerated, stored at 4 ± 2 °C, and analyzed within 24 h of collection.

### 2.3. Detection of Listeria spp.

Detection of *Listeria* spp. in samples was performed using the impedance method, an alternative to the reference ISO 11290-1:2017 standard [30,32].

Samples were homogenized in a Stomacher 400 Circulator at 260 rpm for 2 min with 225 mL of One Broth Listeria (OB, Oxoid, Hampshire, UK) for product samples (raw materials and final products) and 90 mL for sponge samples (including swabs when used in sampling). Samples were incubated at 30 °C for 24 h (pre-enrichment) [32].

After incubation, 1 mL of the pre-enrichment was transferred into 9 mL of OB and placed in a specific two-electrode cell (SY-LAB Geräte GmbH, Purkersdorf, Austria). The impedance change (M-value) was monitored using a µTrac 4200 instrument (SY-LAB Geräte GmbH). The assay was conducted at 30 °C for 48 h (enrichment). Detection was adjusted to an M-value threshold of 5% to minimize background noise. The result was considered positive for *Listeria* spp. when a characteristic impedance curve was observed and the selected threshold was reached [32,33].

### 2.4. Isolation and Identification

Positive samples were plated on two chromogenic agars: Oxoid Chromogenic Listeria Agar (OCLA) (Oxoid) and Rapid L. mono (Bio-Rad, Hercules, CA, USA). Plates were incubated at 37 °C for 48 and 24 h, respectively.

After incubation, characteristic colonies were selected from each agar and subcultured on Tryptone Soy Agar (TSA) (Oxoid). On OCLA, *L. monocytogenes* colonies appear blue/green surrounded by an opaque halo, while other *Listeria* spp. are blue/green colonies without halos. *L. monocytogenes* colonies on Rapid L. mono agar are blue, *L. ivanovii* forms blue-green colonies with yellow halos, and other *Listeria* spp. appear as white colonies. Presumptive *L. monocytogenes* colonies were biochemically confirmed by the rhamnose fermentation test. Several isolates of *L. monocytogenes* were selected from positive samples in which different morphologies were differentiated.

### 2.5. L. monocytogenes Typing

#### 2.5.1. Serotype Identification

Molecular serotyping of *L. monocytogenes* isolates was performed using two multiplex PCR reactions. These PCRs employed five primer pairs (Table 1) specific for the five main *L. monocytogenes* serotypes (1/2a, 1/2b, 1/2c, 4a, and 4b), plus one primer pair specific for *Listeria* spp. [2,3,6].

Briefly, each isolate was plated on TSA and incubated for 24 h at 37 °C. A single colony was then inoculated onto Tryptone Soya Broth (TSB) (Oxoid) and incubated overnight at 37 °C for 14–16 h. For DNA extraction, 1 mL of the overnight culture was centrifuged at 12,000 rpm for 2 min in a Microfuge^®^ 18 Centrifuge (Beckman Coulter^TM^, Brea, CA, USA). The pellet was resuspended in 100 μL of PrepMan^TM^ Ultra Sample Preparation Reagent (Thermo Fisher Scientific, Waltham, MA, USA) and vortexed for 10–30 s. The sample was then incubated at 100 °C for 10 min in a Termoblock Stuart^TM^ Block Heater (Fisher Scientific, MA, USA), cooled to room temperature for 2 min, and centrifuged again at 12,000 rpm for 2 min.

DNA quality (A_260/280_ and A_260/230_) was assessed using the NanoDrop 2000 spectrophotometer (Thermo Fisher Scientific). DNA amplification was performed on a MiniOpticon^TM^ Real-Time PCR System thermal cycler (Bio-Rad).

#### 2.5.2. Pulsed-Field Gel Electrophoresis Typing

Pulsed-field gel electrophoresis (PFGE) was conducted according to the standardized PulseNet protocol for *L. monocytogenes* typing [34]. Genomic DNA was digested with the restriction enzymes *Asc*I and *Apa*I (New England Biolabs, Beverly, MA, USA), and PFGE was performed using the CHEF-DR^®^ III System (Bio-Rad).

Composite PFGE types were obtained by combining the banding patterns from *Asc*I and *Apa*I digestions and designated with an ‘S’, followed by a number identifying the *Asc*I profile. When multiple *Apa*I profiles corresponded to a single *Asc*I profile, the *Asc*I number was followed by a hyphen and a second number for the *Asc*I profile [34,35].

Image acquisition was performed using GelDoc XR equipment (Bio-Rad), and band patterns were analyzed with Quantity One v.4.4.0 software (Bio-Rad). Comparative analysis of PFGE profiles was conducted with Gel Compar^®^ II v 6.5.0 software (Applied Maths, Sint-Martens-Latem, Belgium). Dendrograms of the PFGE profiles, generated from both enzymes, were constructed using the unweighted pair grouping method with arithmetic means (UPGMA) and the Dice similarity coefficient, with an optimization and tolerance level of 1% [33,36]. This process was carried out with the isolates from Industry A and Industry B, both separately by industry and jointly by combining isolates from both industries.

Following established criteria [21,33,36,37,38], isolates with ≥95% similarity were considered to be the same pulsotype. Pulsotypes showing more than 80% similarity were grouped into the same pulsogroup, while those with ≥70% similarity were classified as belonging to the same cluster. Strains with the same PFGE profile isolated repeatedly (at least 3 times) from the environment or equipment for at least 3 months were considered persistent [36].

### 2.6. Statistical Analysis

The chi-square test or Fisher’s exact test, depending on the sample size, was used to compare the contamination present between the different sample categories analyzed: raw materials, final products, processing zones or production lines, operational and pre-operational FCSs, and FCSs and NFCSs. On the one hand, this statistical comparison was conducted within each industry to determine the relevance or importance of each sample category in the contamination routes. On the other hand, it was performed between the two analyzed industries to assess the importance of monitoring and controlling this pathogen in an industry subject to microbiological standards versus one that is not. All analyses were performed with Statgraphics 19 (Version 19.6.05) with a confidence level of 95%. Strain diversity in the two industries was evaluated using Simpson’s diversity index [39].

## 3. Results

### 3.1. Prevalence of Listeria spp. and L. monocytogenes

#### 3.1.1. Industry A

**Figure 2:**
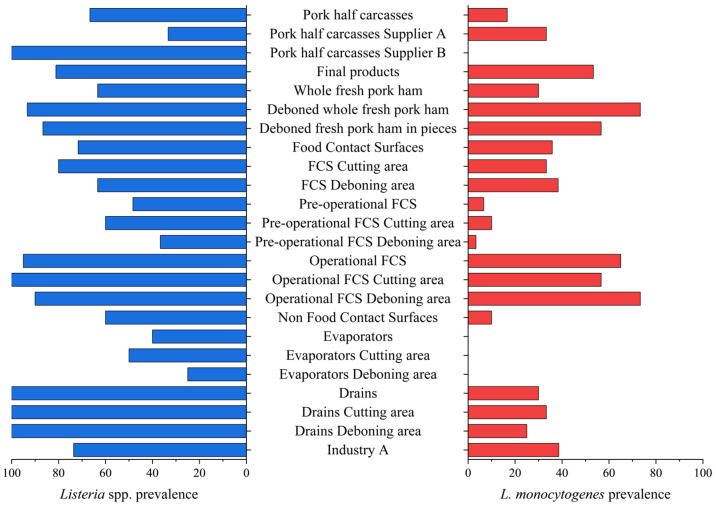
Occurrence of *Listeria* spp. and *L. monocytogenes* in the different Industry A sampling points. The sample category “food contact surface” is abbreviated as “FCS”.

The distribution and prevalence of *Listeria* spp. and *L. monocytogenes* in the samples collected are summarized in Table 2 and Figure 2.

In Industry A, *Listeria* spp. was detected in all sample categories analyzed, with an average prevalence of 75%. The prevalence increased along the processing chain, rising from 66.7% in the raw materials to 81.1% in the final products, although this difference was not statistically significant. The prevalence increased significantly with handling among the three final products analyzed: whole fresh pork hams (FH) (63.3%), deboned whole fresh pork hams (DFH) (93.3%), and deboned fresh pork hams in pieces (DFHP) (86.7%) (*p* < 0.05, chi-square test).

Contamination of food contact surfaces (FCSs) was significantly higher after processing (operational, OP) than after C&D (pre-operational, PO) (95% vs. 48.3%) (*p* < 0.05, chi-square test), being detected on all surfaces analyzed at least once in both cases. A statistically significant difference in the presence of *Listeria* spp. was observed between the FCSs in the common processing area (cutting) and those in the deboning area (80.0% vs. 63.3%, respectively) (*p* < 0.05, chi-square test). This difference was not significant between the two processing areas in either pre-operational or operational FCSs.

Regarding the NFCSs, *Listeria* spp. was detected in 70% of the samples. Specifically, *Listeria* spp. was detected on 40% of the evaporator samples and 100% of the drain samples. No statistically significant differences were observed between FCSs (71.7%) and NFCSs (70%) in terms of *Listeria* spp. prevalence.

Concerning *L. monocytogenes*, it was detected in 40.3% of the total samples analyzed. The pathogen’s prevalence in raw materials was relatively low, with 16.7% of samples testing positive. Contamination increased as the production process progressed, along with *Listeria* spp., reaching 53.3% in the final products, although this difference was not statistically significant. In the final products, *L. monocytogenes* was detected in 30% of the FH samples, 73.3% of the DFH samples, and 56.7% of the DFHP samples. A statistically significant difference was observed between deboned hams (DFH and DFHP) and FH (*p* < 0.05, chi-square test), which may be associated with the greater handling of deboned hams.

For FCSs, *L. monocytogenes* contamination was significantly higher in OP than in PO conditions (65% vs. 6.7%, respectively) (*p* < 0.05, chi-square test). The pathogen was detected at least once on all OP FCSs. Nevertheless, it is important to emphasize that on PO FCSs, it was isolated on the common conveyor belt and nearby metal surfaces, as well as the plastic slat door that comes into contact with the pork hams for deboning. There were no statistically significant differences in the presence of *L. monocytogenes* between FCSs in the common processing area and those in the deboning area, either pre-operational or operational.

*L. monocytogenes* was isolated exclusively from drains (30%) among the NFCS samples. A higher overall prevalence was observed on FCSs (35.8%) compared to NFCSs (15%), although this difference was not statistically significant.

#### 3.1.2. Industry B

**Figure 3:**
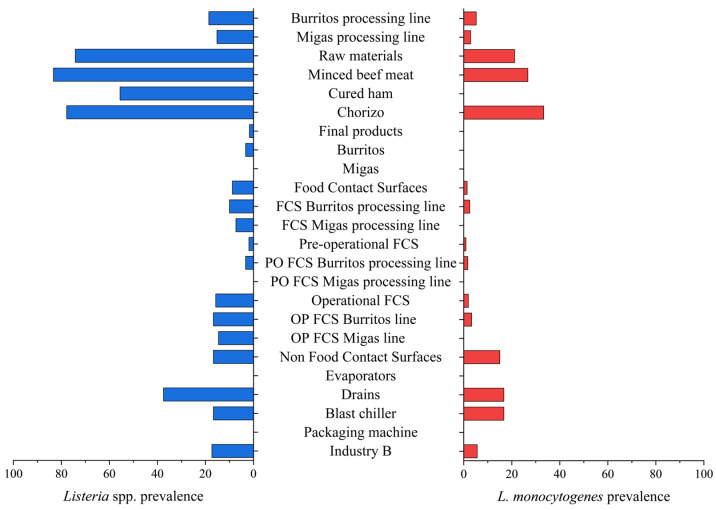
Occurrence of *Listeria* spp. and *L. monocytogenes* in the different Industry B sampling points. The sample categories are abbreviated as “FCS” (food contact surface), “PO” (pre-operational), and “OP” (operational).

The distribution and prevalence of *Listeria* spp. and *L. monocytogenes* in the samples collected are summarized in Table 3 and Figure 3.

In Industry B, *Listeria* spp. was detected in 17.3% of the samples. The highest prevalence was observed in raw materials (74.2%), with statistically significant differences only between fresh minced beef meat (83.3%) and cured ham (55.6%), and not with fresh chorizo (77.8%). The prevalence of *Listeria* spp. significantly decreased with heat treatment (*p* < 0.05, chi-square test), reaching 1.7% in the final products. It was only detected in burritos (3.3%).

Regarding FCSs, *Listeria* spp. was detected in 8.8% of the samples analyzed, decreasing from 15.7% in operational conditions to 1.9% in pre-operational conditions. This reduction was statistically significant (*p* < 0. 05, chi-square test) and was observed in both processing lines. In the burritos processing line, a significant reduction was observed from 16.7% in OP (beef mincer and stainless steel tray [zone C]) to 3.3% in PO (stuffer and stainless steel tray [zone D]) (*p* < 0.05, Fisher’s exact test), with an overall prevalence in FCSs of 10%. In the migas processing line, a significant reduction from 14.6% (cured ham and chorizo mincer, stainless steel tray [zone C], and plastic product storage boxes) to not detected was observed (*p* < 0.05, Fisher’s exact test), with an overall prevalence of 7.3%. No significant differences were observed in the presence of *Listeria* spp. on FCSs, either PO or OP, between the two processing lines.

Concerning NFCSs, the prevalence of *Listeria* spp. was 16.7% (drains in zones A, C, and D and the blast chiller). No statistically significant differences were observed between the overall prevalence in FCSs (8.8%) and NFCSs (16.7%).

*L. monocytogenes* was detected in 5.6% of the samples analyzed. The highest prevalence was observed in raw materials (21.2%), with significant differences between fresh minced beef meat (26.7%) and cured ham (0%), and between chorizo (33.3%) and cured ham (0%) (*p* < 0.05, Fisher’s exact test). The pathogen was not detected in any final products, representing a statistically significant reduction between raw materials and final products (*p* < 0.05, Fisher’s exact test).

On FCSs, *L. monocytogenes* was isolated only from the burritos processing line, with 3.3% in OP FCSs (beef mincer) and 1.7% in PO FCSs (stainless steel tray [zone D]).

For NFCSs, the pathogen was present in 15% of the samples (drains in zones A, C, and D and the blast chiller). A significant difference in the overall presence of *L. monocytogenes* was observed between FCSs (1.4%) and NFCSs (15%) (*p* < 0.05, Fisher’s exact test).

No statistically significant differences were observed in the overall presence of *Listeria* spp. and *L. monocytogenes* between the two production lines analyzed.

### 3.2. Serotype Identification

#### 3.2.1. Industry A

A total of 170 isolates were analyzed, revealing the presence of the four molecular serotypes most commonly associated with human listeriosis worldwide: 1/2a, 1/2b, 1/2c, and 4b. Most isolates belonged to serotype 1/2a (93.5%), followed by 1/2b (4.7%), 4b (1.2%), and, less frequently, serotype 1/2c (0.6%). Serotype 1/2a was detected across all sample types, while serotype 1/2b was isolated from both operational and pre-operational FCSs and drains. Serotypes 4b and 1/2c were detected exclusively on operational FCSs. Operational FCS samples showed the highest diversity of serotypes.

#### 3.2.2. Industry B

A total of 50 isolates were analyzed, revealing the presence of only two molecular serotypes: 1/2a and 1/2c. Serotype 1/2a was the most frequent (92%). This serotype was detected in raw materials, operational (mincer) and pre-operational (stainless steel tray) FCSs of the burritos line, and NFCSs common to both lines. Serotype 1/2c (8%) was isolated from the raw materials of the burritos line (minced beef meat) and an operational FCS of the burritos line (mincer).

### 3.3. Pulsed-Field Gel Electrophoresis Typing

#### 3.3.1. Industry A

**Figure 4:**
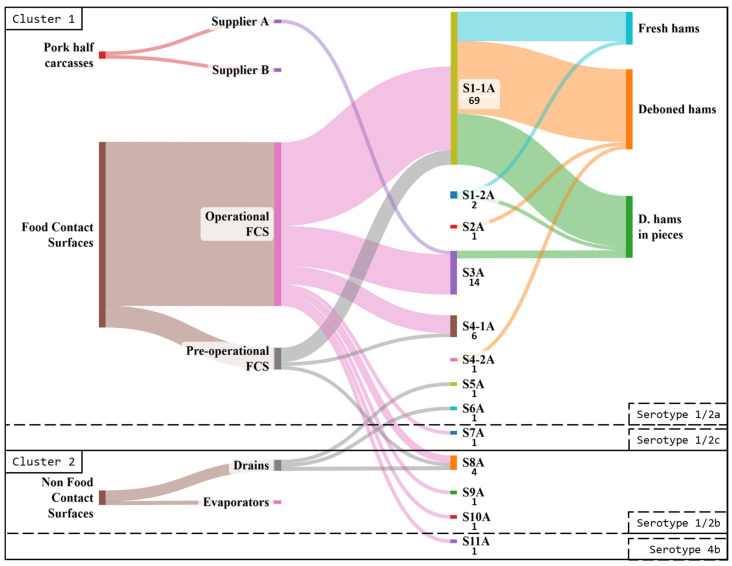
Sankey plot of the distribution of *L. monocytogenes* pulsotypes at the different sampling points in Industry A. The sample category “food contact surface” is abbreviated as “FCS”.

PFGE typing was conducted on 103 selected isolates, representing each positive sample category, sampling date, and serotype. The results revealed 13 different pulsotypes (PTs), classified into two clusters. Cluster 1 included ninety-six isolates of lineage II, belonging to serotypes 1/2a (ninety-five isolates/seven strains) and serotype 1/2c (one isolate/one strain). Cluster 2 comprised seven isolates of lineage I, corresponding to serotypes 1/2b (six isolates/four strains) and serotype 4b (one isolate/one strain) (Figure 4).

Strains with the same PFGE profile that were isolated repeatedly (at least three times) in the environment or equipment for at least 3 months were considered persistent [21]. Four persistent PTs (S1-1A, S3A, S4-1A, and S8A) were identified. Furthermore, two of these PTs were predominant in the industry (representing > 10% of the isolates): S1-1A (67.0%) and S3A (13.6%).

Pulsotype S1-1A (serotype 1/2a) was detected in all analyzed OP FCSs (except the scale) and some PO FCSs (the common conveyor belt, nearby metal surfaces, and the plastic slat door). This pulsotype was detected repeatedly (at least three times) on the two conveyor belts analyzed and nearby metal surfaces, as well as on the set of knives in the deboning area. In addition, it was detected in all final products; however, it was not detected in raw materials.

PT S3A (serotype 1/2a) was isolated in all the common OP FCSs and three out of five of those in the deboning area (plastic slat door, membrane skinner, and storage boxes). It was not detected in PO FCSs. This PT was detected repeatedly (at least three times) on the plastic slat door of the deboning area. S3A was detected in the raw materials and in one of the final products (DFHP).

Pulsotypes S4-1A (serotype 1/2a) and S8A (serotype 1/2b) were found on the OP conveyor belt (S4-1A was repeatedly found at least three times) and nearby metal-surface FCSs common to all three lines. In addition, PT S4-1A was detected on the common PO conveyor belt and two of the FCSs on the deboned ham line (plastic slat door and knife set). PT S8A was also recovered on a common PO metal surface near the conveyor belt and in the drain of the same room. Neither of these two PTs were detected in the raw materials or the final products.

The diversity observed in Industry A was 0.53 (Simpson’s index). This relatively low value is supported by the predominance of two strains. In the final products, diversity increased with manipulation. The diversity was 0.22 in fresh whole hams, 0.18 in deboned fresh hams, and 0.32 in deboned fresh hams in pieces. Regarding FCSs, the diversity was higher in FCSs in the common processing area than in FCSs in the deboning area (0.74 vs. 0.58). This difference between the processing areas was also observed in OP FCSs (0.76 vs. 0.60) and in PO FCSs (0.70 vs. 0.0). Concerning NFCSs, specifically drains, the diversity found was very high (1.0).

#### 3.3.2. Industry B

**Figure 5:**
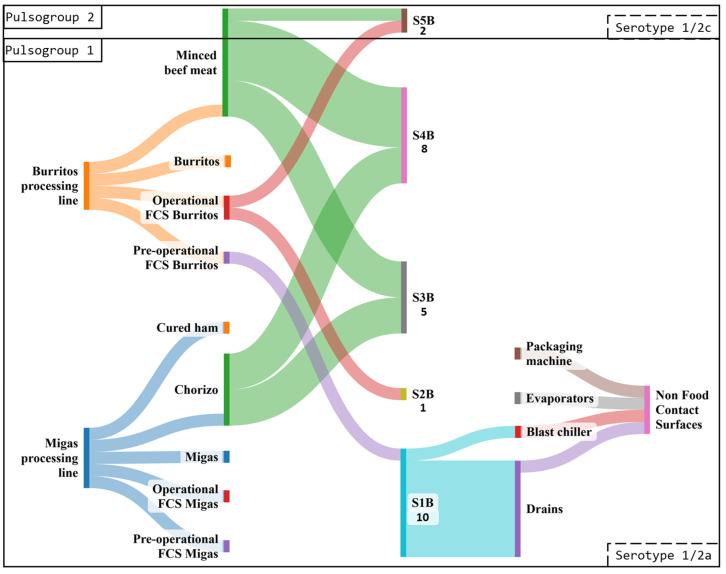
Sankey plot of the distribution of *L. monocytogenes* pulsotypes at the different sampling points in Industry B. The sample category “food contact surface” is abbreviated as “FCS”.

PFGE typing was performed on 26 selected isolates, representing each positive sample category, sampling date, and serotype. Five different pulsotypes were detected and classified into two pulsogroups. Pulsogroup 1 (serotype 1/2a), mostly isolated from NFCSs, included eleven isolates belonging to strains S1B (ten isolates) and S2B (one isolate). Pulsogroup 2, detected mainly in raw materials, comprised fifteen isolates belonging to strains S3B (five isolates, serotype 1/2a), S4B (eight isolates, serotype 1/2a), and S5B (two isolates, serotype 1/2c) (Figure 5).

Strains with the same PFGE profile isolated repeatedly (at least three times) from the environment or equipment for at least 3 months were considered persistent [21]. One persistent strain (S1B) was identified. In addition, three PTs were considered predominant in the industry (representing >10% of isolates): S1B (38.5%), S4B (30.8%), and S3B (19.2%).

Pulsotype S1B (serotype 1/2a) was detected on an FCS in the burritos processing line (pre-operational stainless steel tray), in the blast chiller, and in the drains of zones A, C, and D. Specifically, this strain was isolated in the drain of zone C (where heat treatment is performed) up to five times over 6 months. Pulsotypes S3B and S4B (serotype 1/2a) were repeatedly detected in fresh raw materials (minced beef meat and chorizo).

The diversity observed in industry B was 0.74 (Simpson’s index). This relatively high value is supported by the small number of strains observed. Similar diversity was detected in both processing lines (0.69 for burritos and 0.68 for the migas processing line). Moderate diversity was observed in the raw materials (0.58). Maximum diversity was observed for FCSs (1.0), with the same result in OP (1.0). Nevertheless, the diversity for PO FCSs and NFCSs was null (0.0).

### 3.4. Comparison Between Industries A and B

#### 3.4.1. Prevalence of *Listeria* spp. and *L. monocytogenes*

**Figure 6:**
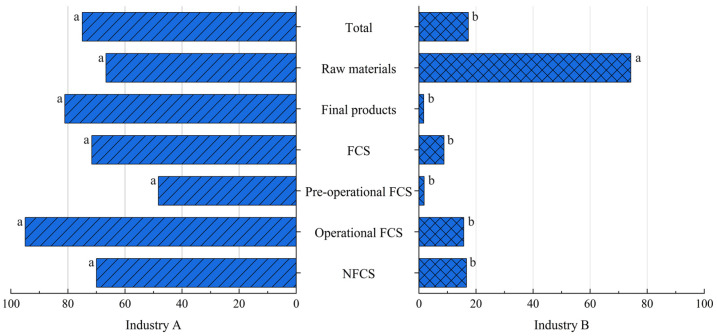
*Listeria* spp. occurrence in Industries A and B according to the sample category. The sample categories are abbreviated as “FCS” (food contact surface) and “NFCS” (non-food contact surface). ^a,b^ Different superscripts within a sample category indicate significant differences (*p* < 0.05).

**Figure 7:**
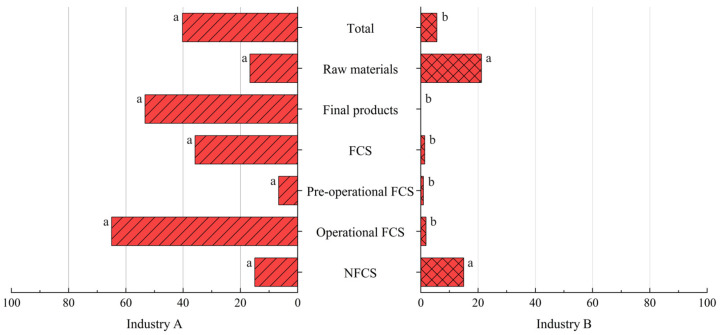
*L. monocytogenes* occurrence in Industries A and B according to the sample category. The sample categories are abbreviated as “FCS” (food contact surface) and “NFCS” (non-food contact surface). ^a,b^ Different superscripts within a sample category indicate significant differences (*p* < 0.05).

In order to evaluate the importance of the surveillance and control of *L. monocytogenes* in the meat industry, a statistical comparison of the contamination present among the different sample categories analyzed was carried out in an industry subject to microbiological standards versus another that is not. The distribution of *Listeria* spp. and *L. monocytogenes* prevalence in both industries in the different sample categories analyzed is shown in Figure 6 and Figure 7, respectively.

Statistically significant differences in the overall presence of *Listeria* spp. and *L. monocytogenes* were observed between the two industries (*p* < 0.05, chi-square test). Regarding raw materials, no statistically significant differences were observed in the presence of either *Listeria* spp. or *L. monocytogenes*. The occurrence of *Listeria* spp. and pathogenic species was statistically higher in the final products and FCSs and OP FCSs from Industry A compared to those from Industry B (*p* < 0.05, chi-square test). Concerning PO FCSs, this difference was also significant for both *Listeria* spp. (*p* < 0.05, chi-square) and *L. monocytogenes* (*p* < 0.05, Fisher’s exact test). For NFCSs, this difference was significant only for *Listeria* spp. (*p* < 0.05, chi-square test) and was not significant for pathogen presence.

#### 3.4.2. Pulsed-Field Gel Electrophoresis Typing

The banding patterns obtained by isolates from both industries were compared. It was observed that strain S2B from Industry B (operational mincer of the burrito processing line) presented a similarity of ≥90% with strain S1A from Industry A (predominant and persistent). Furthermore, strains S2A (deboned fresh ham in pieces) and S7A (metal surfaces near the common conveyor belt) from Industry A coincided with strains S1B (pre-operational stainless steel tray on the burrito processing line, drains, and the blast chiller) and S5B (minced beef meat and beef mincer) from Industry B, respectively. The resulting dendrogram is shown in Supplemental Figure S1.

## 4. Discussion

The control of *L. monocytogenes* in meat processing facilities remains a significant challenge due to the ongoing introduction of the microorganism into plants, as well as the persistence of strains in the environment. The objective of this study was to identify and evaluate the importance of *L. monocytogenes* contamination routes, to evaluate the relevance of persistence in the risk of contamination for its control, and to evaluate the importance of surveillance and control of *L. monocytogenes* in an industry dedicated to pork cutting and an industry dedicated to processing RTE foods with pork and beef meat ingredients.

The general prevalence of *L. monocytogenes* in Industry A (pork cutting) (40.3%), particularly the pronounced increase along the processing chain (from 16.7% in the raw materials to 53.3% in the final products), underscores the significant risk of cross-contamination during processing. These findings align with previous studies reporting a low to moderate prevalence of the pathogen in pork carcasses (0–22%) [23,38], but a higher prevalence in fresh whole hams [24,40]. A significant difference in *L. monocytogenes* contamination was observed between operational (OP) (65%) and pre-operational (PO) (6.7%) food contact surfaces (FCSs). This suggests that processing activities contribute significantly to contamination. This contamination rate mirrors findings from Rugna et al. (2021) [8] in the trimming zone of two ham processing plants in Italy, though it is higher than those observed in Romania by Sala et al. (2016) [41].

Regarding Industry B (RTE foods), the overall prevalence *L. monocytogenes* was low (5.6%), highlighting fresh meat raw materials (21.2%) and non-food contact surfaces (NFCSs) (15%). A low overall prevalence was observed for FCSs (1.4%), being slightly higher in OP (1.9%) than in PO (0.9%). Previous studies observed a higher overall prevalence (between 9% and 19.4%), as well as for operational FCSs (between 7.7% and 24.8%) in cured ham plants [24,27]. The contamination rate of fresh meat raw materials (21.2%) was similar to that observed by Thévenot et al. (2006) [42] in sausage processing plants.

A higher prevalence of *Listeria* spp. compared to *L. monocytogenes* was observed in all sample categories analyzed in both industries, even being detected in RTE products (burritos). The trend in *Listeria* spp. contamination was the same as that observed for the pathogenic species, being higher in those sample categories that presented higher *L. monocytogenes* contamination. This observation aligns with previous studies that have reported similar trends in food processing environments, demonstrating the importance of monitoring *Listeria* spp. as part of a comprehensive food safety strategy [37,41].

Significantly higher overall prevalence rates of both *Listeria* spp. and *L. monocytogenes* were observed in Industry A (pork cutting) compared to Industry B (RTE foods). While raw materials showed similar contamination levels, Industry A exhibited an increase in prevalence along the processing chain. In contrast, Industry B maintained significantly lower contamination levels. Moreover, an absence of contamination spread from raw materials to FCSs was also observed in Industry B. These results might be attributed to the heat treatments applied to Industry B products, as well as stricter compliance with the microbiological criteria [15]. This aligns with the findings of Rugna et al. (2021) [8], who reported a higher prevalence of *L. monocytogenes* in pig slaughterhouses and processing plants, especially in fresh hams. These results highlight the critical role of the processing environment and handling practices in *Listeria* control, emphasizing the need for comprehensive strategies to prevent cross-contamination and persistence in meat processing environments.

In both industries, a significant reduction in the presence of *Listeria* spp. was observed after cleaning and disinfection (C&D). However, *L. monocytogenes* was detected on at least one FCS in each industry after C&D protocols. This suggests that, at certain critical points, the effectiveness of the applied protocols may be insufficient, or recontamination may occur after C&D protocols. According to previous studies, the persistence of *L. monocytogenes* in food processing environments has been attributed to a combination of factors, including inefficient C&D procedures and suboptimal equipment design [8,26,38].

Previous studies have identified NFCSs as sources of contamination and dissemination of *L. monocytogenes*. In Industry A, a 30% prevalence was detected in drains, with a persistent strain (S8A) identified. The pathogen was detected in 18.8% of the NFCSs in Industry B, with the only persistent strain in this industry (S1B) being identified in the drains of three distinct zones. These results confirm the necessity for adequate sanitation procedures to prevent these areas from becoming significant contamination sources, as well as to monitor these surfaces to have a complete picture of the contamination present in the industry.

In the fresh meat processing environment (Industry A), two predominant and persistent strains were identified. Strain S3A, the only one detected in pork carcasses, was predominant and persistent in the processing environment throughout the sampling year. This strain was only detected once in one of the final products. Nevertheless, strain S1-1A, which was predominant and persistent in the processing environment, was detected repeatedly in all three final products tested and not in any of the raw material samples analyzed. Previous studies observed minimal *L. monocytogenes* transfer from primary production to processed products [40] and highlighted a transfer from surfaces to products [8,23]. Moreover, this is corroborated by the low strain diversity detected in the final products (between 0.18 and 0.32).

A high prevalence of *L. monocytogenes* was detected in Industry A’s operational FCSs, with a limited number of persistent strains identified. This behavior is widely described [8,23,37]. The presence of all persistent strains on the common conveyor belt and/or nearby metal surfaces is noteworthy, as these facilities have shown to be of critical importance in several studies [8,23,43].

In the context of the RTE food processing environment (Industry B), one predominant and persistent strain was identified (S1B). Strain S1B was persistently detected in the drains of three zones, both pre-lethal and post-lethal, in the blast chiller, and on a post-lethal PO FCS. In addition, null strain diversity was observed on the NFCSs, highlighting the persistence of a single strain on these surfaces. This finding underscores the importance of NFCSs as contamination and dissemination sources of pathogens in the industry. Furthermore, these results underscore the importance of monitoring these surfaces as indicator sites of contamination present in the industry environment.

Strains S3B and S4B were detected exclusively in fresh meat raw materials, with no transfer into the processing environment. These results are supported by other studies indicating that the transfer of *L. monocytogenes* from primary production to processed products is minimal [40]. This underscores the importance of monitoring the processing environment and the need for effective C&D protocols to ensure the food safety of the final products.

Our findings confirm the ability of *L. monocytogenes* to survive and persist in the same ecological niche, becoming endemic in the processing environment [29,37]. The predominance and persistence of a small number of strains in both industries point to the need for specific measures to control the pathogen in the industry. Furthermore, this highlights the importance of the molecular characterization of isolates by pulsed-field electrophoresis (PFGE) in order to identify the contamination sources or niches present in the environment, to trace the contamination sources in the production chain, and to evaluate the effectiveness of the implemented control strategies.

Three strains with the same PFGE profile were detected in two industries with significantly different environments. One of the industries is dedicated to pork cutting, while the other specializes in the production of RTE foods. The presence of genetically similar strains in such different environments suggests the possible existence of common contamination vectors and the ability of certain strains to adapt and persist in the food processing environment. Similar results were reported by Ortiz et al. (2016) [26], who identified the same persistent *L. monocytogenes* strains from geographically separated pork processing plants. Furthermore, this genetic similarity between industries handling different meat products highlights the adaptability and persistence of certain *L. monocytogenes* strains in diverse food processing environments, as noted by Rugna et al. (2021) [8] in their study on the distribution of *L. monocytogenes* in slaughterhouses and pork processing plants.

All persistent strains detected in both industries, except one, belong to serotype 1/2a (lineage II). Serotype 1/2a was the most prevalent across all facilities analyzed in both industries (93.5% in Industry A and 92% in Industry B). Other previous studies have pointed to this serotype as the dominant in pork processing environments [37,44]. Strains of this serotype have demonstrated a higher biofilm-forming capacity compared to lineage I [9]. Notably, serotype 1/2a has been associated with epidemic cases of listeriosis in Canada [45]. Serotypes 1/2b (4.7% in Industry A), 4b (1.2% in Industry A), and 1/2c (0.6% in Industry A and 8% in Industry B) were also detected less frequently. Serotype 4b includes highly virulent strains linked to large outbreaks of listeriosis [46,47].

The findings highlight that traditional detection methods by themselves do not provide enough information on the epidemiology of *L. monocytogenes* as they do not distinguish between sporadic and persistent contamination events. The molecular characterization of strains by using PFGE has proven to be essential for monitoring the presence and behavior of *L. monocytogenes* in food processing environments. It enables the identification of critical surfaces or facilities that are difficult to access or challenging to clean and disinfect, as well as potential transmission routes and the presence of persistent strains, which is crucial for enhancing hygiene protocols. Therefore, these findings underscore the importance of the molecular characterization of *L. monocytogenes* contamination for designing and implementing specific prevention and control strategies in meat processing environments.

In conclusion, the more exhaustive control of the presence of *L. monocytogenes* in RTE food industries is reflected in a clear reduction in the pathogen’s prevalence in the final products. This highlights the effectiveness of robust monitoring and intervention strategies. Extending these measures to fresh meat industries could improve their overall hygiene conditions. In addition, the importance of comprehensive surveillance, including the detection and characterization of contamination in indicator sites, is emphasized as a key step in the prevention and control of *L. monocytogenes* in processing environments. Equally crucial is the consideration of plant and equipment designs to minimize the risk of pathogen persistence, ensuring safer food production practices.

## Figures and Tables

**Table 1:** Primers used in this study and their sequences.

Gene	Primer Sequences (5′–3′)	Product Size (pb)	Reference
*lmo*1118	F: AGG GGT CTT AAA TCC TGG AA R: CGG CTT GTT CGG CAT ACT TA	906	[2]
*lmo*0737	F: AGG GCT TCA AGG ACT TAC CC R: ACG ATT TCT GCT TGC CAT TC	691	[2]
*ORF2110*	F: AGT GGA CAA TTG ATT GGT GAA R: CAT CCA TCC CTT ACT TTG GAC	597	[2]
*ORF2819*	F: AGC AAA ATG CCA AAA CTC GT R: CAT CAC TAA AGC CTC CCA TTG	471	[2]
*prs*	F: GCT GAA GAG ATT GCG AAA GAA G R: CAA AGA AAC CTT GGA TTT GCG G	370	[2]
*prfA*	F: GAT ACA GAA ACA TCG GTT GGC R: GTG TAA TCT TGA TGC CAT CAG G	274	[3]
*flaA*	F: TTA CTA GAT CAA ACT GCT CC R: AAG AAA AGC CCC TCG TCC	538	[6]

**Table 2:** Distribution of samples positive for *Listeria* spp. and *L. monocytogenes* in the Industry A sampling points.

Sample Category	No. of Samples	*Listeria* Species No. of Positive Samples (%)	
		*Listeria* spp.	*L. monocytogenes*
Pork half carcasses	6	4 (66.7)	1 (16.7)
Pork half carcasses, Supplier A	3	1 (33.3)	1 (33.3)
Pork half carcasses, Supplier B	3	3 (100.0)	0 (0.0)
Final products	90	73 (81.1)	48 (53.3)
Whole fresh pork ham	30	19 (63.3) ^a^	9 (30.0) ^a^
Deboned whole fresh pork ham	30	28 (93.3) ^b^	22 (73.3) ^b^
Deboned fresh pork ham in pieces	30	26 (86.7) ^ab^	17 (56.7) ^b^
Total FCSs	120	86 (71.7)	43 (35.8)
FCSs cutting area	60	48 (80.0) ^a^	20 (33.3) ^a^
FCSs deboning area	60	38 (63.3) ^b^	23 (38.3) ^a^
Pre-operational FCSs	60	29 (48.3)	4 (6.7)
PO FCSs cutting area	30	18 (60.0) ^a^	3 (10.0) ^a^
PO FCSs deboning area	30	11 (36.7) ^a^	1 (3.3) ^a^
Operational FCSs	60	57 (95.0)	39 (65.0)
OP FCSs cutting area	30	30 (100.0) ^a^	17 (56.7) ^a^
OP FCSs deboning area	30	27 (90.0) ^a^	22 (73.3) ^a^
Total NFCSs	20	14 (70.0)	3 (15.0)
Evaporators	10	4 (40.0)	0 (0.0)
Evaporators cutting area	6	3 (50.0)	0 (0.0)
Evaporators deboning area	4	1 (25.0)	0 (0.0)
Drains	10	10 (100.0)	3 (30.0)
Drains cutting area	6	6 (100.0)	2 (33.3)
Drains deboning area	4	4 (100.0)	1 (25.0)
**Industry A**	**236**	**177 (75.0)**	**95 (40.3)**

^a,b^ Different superscripts within a sample category and column indicate significant differences (*p* < 0.05). The sample categories are abbreviated as “FCS” (food contact surface), “PO” (pre-operational), “OP” (operational) and “NFCS” (non-food contact surface).

**Table 3:** Distribution of samples positive for *Listeria* spp. and *L. monocytogenes* in the Industry B sampling points.

Sample Category	No. of Samples	*Listeria* Species No. of Positive Samples (%)	
		*Listeria* spp.	*L. monocytogenes*
Burritos processing line	210	39 (18.6) ^a^	11 (5.2) ^a^
Migas processing line	192	31 (16.1) ^a^	6 (3.1) ^a^
Raw materials	66	49 (74.2)	14 (21.2)
Minced beef meat	30	25 (83.3) ^a^	8 (26.7) ^a^
Cured ham	18	10 (55.6) ^b^	0 (0.0) ^b^
Chorizo	18	14 (77.8) ^ab^	6 (33.3) ^a^
Final products	120	2 (1.7)	0 (0.0)
Burritos	60	2 (3.3)	0 (0.0)
Migas	60	0 (0.0)	0 (0.0)
Total FCSs	216	19 (8.8)	3 (1.4)
FCSs Burritos processing line	120	12 (10.0) ^a^	3 (2.5) ^a^
FCSs Migas processing line	96	7 (7.3) ^a^	0 (0.0) ^a^
Pre-operational FCSs	108	2 (1.9)	1 (0.9)
PO FCSs Burritos processing line	60	2 (3.3) ^a^	1 (1.7) ^a^
PO FCSs Migas processing line	48	0 (0.0) ^a^	0 (0.0) ^a^
Operational FCSs	108	17 (15.7)	2 (1.9)
OP FCSs Burritos processing line	60	10 (16.7) ^a^	2 (3.3) ^a^
OP FCSs Migas processing line	48	7 (14.6) ^a^	0 (0.0) ^a^
Total NFCSs	60	10 (16.7)	9 (15.0)
Evaporators	24	0 (0.0)	0 (0.0)
Drains	24	9 (37.5)	8 (33.3)
Blast chiller	6	1 (16.7)	1 (16.7)
Packaging machine	6	0 (0.0)	0 (0.0)
**Industry B**	**462**	**80 (17.3)**	**26 (5.6)**

^a,b^ Different superscripts within a sample category and column indicate significant differences (*p* < 0.05). The sample categories are abbreviated as “FCS” (food contact surface), “PO” (pre-operational), “OP” (operational) and “NFCS” (non-food contact surface).

## Data Availability

The original contributions presented in this study are included in the article/Supplementary Materials. Further inquiries can be directed to the corresponding author.

## Supplementary Materials

The following supporting information can be downloaded at: https://www.mdpi.com/article/10.3390/foods14091519/s1, Table S1: Description of sampling points in Industry A; Table S2: Description of sampling points in Industry B; Figure S1. Dendrogram obtained from the *Asc*I restriction of isolates from Industries A and B.

## Abbreviations

The following abbreviations are used in this manuscript:

EU	European Union
RTE	Ready-To-Eat
GDP	Spanish Gross Domestic Product
C&D	Cleaning and Disinfection
FCS	Food Contact Surfaces
NFCS	Non-Food Contact Surfaces
OB	One Broth Listeria
OCLA	Oxoid Chromogenic Listeria Agar
TSA	Tryptone Soy Agar
TSB	Tryptone Soya Broth
PFGE	Pulsed-Field Gel Electrophoresis
FH	Whole Fresh Pork Hams
DFH	Deboned Whole Fresh Pork Hams
DFHP	Deboned Fresh Pork Hams In Pieces
OP	Operational
PO	Pre-Operational
PT	Pulsotype
