# Supplementary material for: Control of Persistent Listeria monocytogenes in the Meat Industry: From Detection to Prevention
Source: Foods. 2025 Apr 26;14(9):1519. doi: 10.3390/foods14091519 (PMC12071965; doi:10.3390/foods14091519)
Supplement: Supplementary file 1 [file foods-14-01519-s001.zip › foods-3587147-supplementary.pdf]

Table S1. Description of sampling points in Industry A.

Line	Sample type	Sample point	Description	Sample location	Activity	Sampling time	Sample number	
	Raw materials	1	Pork half carcasses Supplier A	Zone A	Reception	Start of processing	3*	6
		2	Pork half carcasses Supplier B				3*	
	Final products	3	Whole fresh hams	Zone B	Cutting	End of processing	30	90
		4	Deboned whole fresh hams	Zone C	Deboning		30	
		5	Deboned fresh hams in pieces				30	
Common to all three lines	FCS	6	Stainless steel guard	Zone B	Cutting	PO	6	60
						OP	6	
		7	Scale			PO	6	
						OP	6	
		8	Conveyor belt			PO	6	
						OP	6	
		9	Knife, glove, and knife sharpener set			PO	6	
						OP	6	
		10	Stainless steel rollers and table			PO	6	
						OP	6	
	NFCS	11	Evaporator	Zone A	Reception	-	2	6
		12		Zone B	Cutting	-	2	
		13		Zone D	Storage	-	2	
		14	Drain	Zone A	Reception	-	2	6
		15		Zone B	Cutting	-	2	
		16		Zone D	Storage	-	2	
Deboned fresh ham lines	FCS	17	Plastic slat door	Zone C	Storage	PO	6	60
						OP	6	
		18	Conveyor belt		Deboning	PO	6	
						OP	6	
		19	Knife, glove, and knife sharpener set			PO	6	
						OP	6	
		20	Membrane skinner			PO	6	
						OP	6	
		21	Plastic product storage boxes			PO	6	
						OP	6	
	NFCS	22	Evaporator		Storage	-	2	4
		23			Deboning	-	2	
		24	Drain		Storage	-	2	4
		25			Deboning	-	2	

*Each sample includes 5 pork half carcasses.
The sample categories are abbreviated as "FCS" (food contact surface), "NFCS" (non-food contact surface), "OP" (operational), and "PO" (pre-operational).

Table S2. Description of sampling points in Industry B.

Line	Sample type	Sample point	Description	Sampling area	Sample location	Activity	Sampling time	Sample number	
Burritos processing line	Raw materials	1	Fresh minced beef meat	Pre-lethal	Zone B	Cutting	Start of processing	30	30
	Final products	2	Burritos	Post-lethal	Zone F	Storage	End of processing	60	60
	FCS	3	Beef mincer	Pre-lethal	Zone B	Cutting	PO	12	120
							OP	12	
		4	Stainless steel tray	Post-lethal	Zone C	Heat treatment	PO	12	
							OP	12	
		5	Stainless steel tray		Zone D	Handling	PO	12	
							OP	12	
		6	Stuffer				PO	12	
							OP	12	
		7	Stainless steel table				PO	12	
							OP	12	
Migas processing line	Raw materials	8	Cured ham	Pre-lethal	Zone A	Storage	Start of processing	18	36
		9	Chorizo				Start of processing	18	
	Final products	10	Migas	Post-lethal	Zone F	Storage	End of processing	60	60
	FCS	11	Plastic product storage boxes	Pre-lethal	Zone A	Storage	PO	12	96
							OP	12	
		12	Plastic trolley				PO	12	
							OP	12	
		3	Cured ham and chorizo mincer		Zone B	Cutting	PO	12	
							OP	12	
		13	Stainless steel tray	Post-lethal	Zone C	Heat treatment	PO	12	
							OP	12	
Common to both lines	NFCS	14	Evaporator	Pre-lethal	Zone A	Storage	-	6	24
		15			Zone B	Cutting	-	6	
		16		Post-lethal	Zone C	Heat treatment	-	6	
		17			Zone D	Handling	-	6	
		18	Drain	Pre-lethal	Zone A	Storage	-	6	24
		19			Zone B	Cutting	-	6	
		20		Post-lethal	Zone C	Heat treatment	-	6	
		21			Zone D	Handling	-	6	
		22	Blast chiller		Zone C	Heat treatment	-	6	6
		23	Packaging machine		Zone E	Packaging	-	6	6
								**462**	

The sample categories are abbreviated as "FCS" (food contact surface), "NFCS" (non-food con-tact surface), "OP" (operational), and "PO" (pre-operational).

Figure S1. Dendrogram obtained from *Asc*I restriction of isolates from industries A and B.

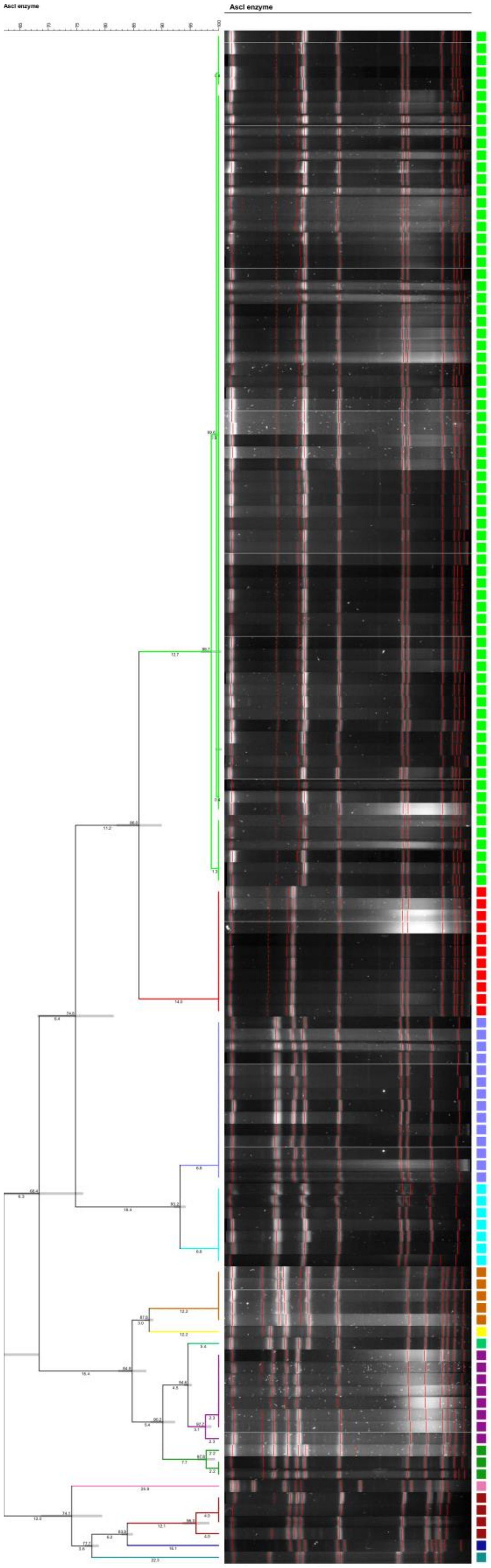

Key	Serotype	Pulsotype	Industry	Processing line	Sample category	Sample area	Sampling zone	Sample location	Sampling date
I315	1/2a	S1	A	Deboned hams	Final Product	Deboning	Zone C	Deboned fresh ham in pieces	18/10/2022
I59	1/2a	S1	A	Deboned hams	Final Product	Deboning	Zone C	Deboned fresh ham in pieces	03/11/2021
I34	1/2a	S1	A	Deboned hams	Pre-operational FCS	Deboning	Zone C	Plastic slat door	05/10/2021
I39	1/2a	S1	A	Whole fresh hams	Final Product	Cutting	Zone B	Whole fresh ham	03/11/2021
I51	1/2a	S1	A	Deboned hams	Final Product	Deboning	Zone C	Deboned whole fresh ham	03/11/2021
I30	1/2a	S1	A	Common	Pre-operational FCS	Cutting	Zone B	Conveyor belt	05/10/2021
I10	1/2a	S1	A	Common	Operational FCS	Cutting	Zone B	Knife, glove, and knife sharpener set	05/10/2021
I116	1/2a	S1	A	Deboned hams	Operational FCS	Deboning	Zone C	Membrane skinner	25/01/2022
I119	1/2a	S1	A	Deboned hams	Operational FCS	Deboning	Zone C	Plastic product storage boxes	25/01/2022
I13	1/2a	S1	A	Common	Operational FCS	Cutting	Zone B	Stainless steel rollers and table	05/10/2021
I131	1/2a	S1	A	Whole fresh hams	Final Product	Cutting	Zone B	Whole fresh ham	08/03/2022
I135	1/2a	S1	A	Deboned hams	Final Product	Deboning	Zone C	Deboned whole fresh ham	08/03/2022
I138	1/2a	S1	A	Deboned hams	Final Product	Deboning	Zone C	Deboned whole fresh ham	08/03/2022
I141	1/2a	S1	A	Deboned hams	Final Product	Deboning	Zone C	Deboned whole fresh ham	08/03/2022
I148	1/2a	S1	A	Deboned hams	Final Product	Deboning	Zone C	Deboned fresh ham in pieces	08/03/2022
I150	1/2a	S1	A	Deboned hams	Final Product	Deboning	Zone C	Deboned fresh ham in pieces	08/03/2022
I161	1/2a	S1	A	Deboned hams	Final Product	Deboning	Zone C	Deboned whole fresh ham	15/03/2022
I163	1/2a	S1	A	Deboned hams	Final Product	Deboning	Zone C	Deboned whole fresh ham	15/03/2022
I165	1/2a	S1	A	Deboned hams	Final Product	Deboning	Zone C	Deboned whole fresh ham	15/03/2022
I167	1/2a	S1	A	Deboned hams	Final Product	Deboning	Zone C	Deboned whole fresh ham	15/03/2022
I172	1/2a	S1	A	Deboned hams	Final Product	Deboning	Zone C	Deboned fresh ham in pieces	15/03/2022
I175	1/2a	S1	A	Deboned hams	Final Product	Deboning	Zone C	Deboned fresh ham in pieces	15/03/2022
I178	1/2a	S1	A	Common	Operational FCS	Cutting	Zone B	Stainless steel guard	05/04/2022
I183	1/2a	S1	A	Common	Operational FCS	Cutting	Zone B	Conveyor belt	05/04/2022
I186	1/2a	S1	A	Common	Operational FCS	Cutting	Zone B	Knife, glove, and knife sharpener set	05/04/2022
I188	1/2a	S1	A	Common	Operational FCS	Cutting	Zone B	Stainless steel rollers and table	05/04/2022
I192	1/2a	S1	A	Deboned hams	Operational FCS	Deboning	Zone C	Plastic slat door	05/04/2022
I195	1/2a	S1	A	Deboned hams	Operational FCS	Deboning	Zone C	Conveyor belt	05/04/2022
I200	1/2a	S1	A	Deboned hams	Operational FCS	Deboning	Zone C	Plastic product storage boxes	05/04/2022
I21	1/2a	S1	A	Deboned hams	Operational FCS	Deboning	Zone C	Knife, glove, and knife sharpener set	05/10/2021
I211	1/2a	S1	A	Common	Pre-operational FCS	Cutting	Zone B	Stainless steel rollers and table	05/04/2022
I214	1/2a	S1	A	Deboned hams	Final Product	Deboning	Zone C	Deboned whole fresh ham	20/04/2022
I217	1/2a	S1	A	Deboned hams	Final Product	Deboning	Zone C	Deboned whole fresh ham	20/04/2022
I220	1/2a	S1	A	Whole fresh hams	Final Product	Cutting	Zone B	Whole fresh ham	20/04/2022
I225	1/2a	S1	A	Whole fresh hams	Final Product	Cutting	Zone B	Whole fresh ham	20/04/2022
I238	1/2a	S1	A	Deboned hams	Final Product	Deboning	Zone C	Deboned fresh ham in pieces	20/04/2022
I248	1/2a	S1	A	Deboned hams	Final Product	Deboning	Zone C	Deboned whole fresh ham	20/04/2022
I260	1/2a	S1	A	Deboned hams	Operational FCS	Deboning	Zone C	Conveyor belt	10/05/2022
I263	1/2a	S1	A	Deboned hams	Operational FCS	Deboning	Zone C	Knife, glove, and knife sharpener set	10/05/2022
I273	1/2a	S1	A	Deboned hams	Final Product	Deboning	Zone C	Deboned whole fresh ham	17/05/2022
I289	1/2a	S1	A	Whole fresh hams	Final Product	Cutting	Zone B	Whole fresh ham	18/10/2022
I292	1/2a	S1	A	Whole fresh hams	Final Product	Cutting	Zone B	Whole fresh ham	18/10/2022
I295	1/2a	S1	A	Whole fresh hams	Final Product	Cutting	Zone B	Whole fresh ham	18/10/2022
I299	1/2a	S1	A	Whole fresh hams	Final Product	Cutting	Zone B	Whole fresh ham	18/10/2022
I301	1/2a	S1	A	Deboned hams	Final Product	Deboning	Zone C	Deboned whole fresh ham	18/10/2022
I304	1/2a	S1	A	Deboned hams	Final Product	Deboning	Zone C	Deboned whole fresh ham	18/10/2022
I307	1/2a	S1	A	Deboned hams	Final Product	Deboning	Zone C	Deboned whole fresh ham	18/10/2022
I309	1/2a	S1	A	Deboned hams	Final Product	Deboning	Zone C	Deboned whole fresh ham	18/10/2022
I313	1/2a	S1	A	Deboned hams	Final Product	Deboning	Zone C	Deboned whole fresh ham	18/10/2022
I318	1/2a	S1	A	Deboned hams	Final Product	Deboning	Zone C	Deboned fresh ham in pieces	18/10/2022
I322	1/2a	S1	A	Deboned hams	Final Product	Deboning	Zone C	Deboned fresh ham in pieces	18/10/2022
I324	1/2a	S1	A	Deboned hams	Final Product	Deboning	Zone C	Deboned fresh ham in pieces	18/10/2022
I328	1/2a	S1	A	Deboned hams	Final Product	Deboning	Zone C	Deboned fresh ham in pieces	18/10/2022
I339	1/2a	S1	A	Common	Operational FCS	Cutting	Zone B	Stainless steel rollers and table	25/10/2022
I341	1/2a	S1	A	Deboned hams	Operational FCS	Deboning	Zone C	Plastic slat door	25/10/2022
I343	1/2a	S1	A	Deboned hams	Operational FCS	Deboning	Zone C	Conveyor belt	25/10/2022
I346	1/2a	S1	A	Deboned hams	Operational FCS	Deboning	Zone C	Knife, glove, and knife sharpener set	25/10/2022
I348	1/2a	S1	A	Deboned hams	Operational FCS	Deboning	Zone C	Membrane skinner	25/10/2022
I350	1/2a	S1	A	Common	Pre-operational FCS	Cutting	Zone B	Stainless steel guard	25/10/2022
I54	1/2a	S1	A	Deboned hams	Final Product	Deboning	Zone C	Deboned whole fresh ham	03/11/2021
I57	1/2a	S1	A	Deboned hams	Final Product	Deboning	Zone C	Deboned whole fresh ham	03/11/2021
I67	1/2a	S1	A	Deboned hams	Final Product	Deboning	Zone C	Deboned fresh ham in pieces	03/11/2021
I7	1/2a	S1	A	Common	Operational FCS	Cutting	Zone B	Conveyor belt	05/10/2021
I77	1/2a	S1	A	Common	Operational FCS	Cutting	Zone B	Conveyor belt	09/11/2021
I89	1/2a	S1	A	Deboned hams	Operational FCS	Deboning	Zone C	Conveyor belt	09/11/2021
P113	1/2a	S1	B	Burritos	Operational FCS	Pre-lethal	Zone B	Beef mincer	19/07/2022
I146	1/2a	S1	A	Deboned hams	Final Product	Deboning	Zone C	Deboned fresh ham in pieces	08/03/2022
I93	1/2a	S1	A	Deboned hams	Operational FCS	Deboning	Zone C	Knife, glove, and knife sharpener set	09/11/2021
I170	1/2a	S1	A	Deboned hams	Final Product	Deboning	Zone C	Deboned whole fresh ham	15/03/2022
I43	1/2a	S1	A	Whole fresh hams	Final Product	Cutting	Zone B	Whole fresh ham	03/11/2021
I63	1/2a	S1	A	Deboned hams	Final Product	Deboning	Zone C	Deboned fresh ham in pieces	03/11/2021
I65	1/2a	S1	A	Deboned hams	Final Product	Deboning	Zone C	Deboned fresh ham in pieces	03/11/2021
I245	1/2a	S2	A	Deboned hams	Final Product	Deboning	Zone C	Deboned whole fresh ham	20/04/2022
P104	1/2a	S2	B	Common	NFCS	Post-lethal	Zone C	Drain	12/07/2022
P107	1/2a	S2	B	Common	NFCS	Post-lethal	Zone C	Drain	12/07/2022
P116	1/2a	S2	B	Common	NFCS	Post-lethal	Zone C	Drain	13/09/2022
P120	1/2a	S2	B	Common	NFCS	Post-lethal	Zone C	Drain	22/11/2022
P127	1/2a	S2	B	Common	NFCS	Post-lethal	Zone C	Drain	13/12/2022
P130	1/2a	S2	B	Common	NFCS	Post-lethal	Zone C	Drain	31/1/2023
P132	1/2a	S2	B	Common	NFCS	Post-lethal	Zone C	Drain	31/1/2023
P138	1/2a	S2	B	Burritos	Pre-operational FCS	Post-lethal	Zone C	Stainless steel tray	14/2/2023
P140	1/2a	S2	B	Common	NFCS	Pre-lethal	Zone A	Drain	14/2/2023
P142	1/2a	S2	B	Common	NFCS	Post-lethal	Zone C	Blast chiller	14/2/2023
I124	1/2a	S3	A	Common	Raw material	Reception	Zone A	Pork half carcasses Supplier A	08/03/2022
I153	1/2a	S3	A	Deboned hams	Final Product	Deboning	Zone C	Deboned fresh ham in pieces	08/03/2022
I181	1/2a	S3	A	Common	Operational FCS	Cutting	Zone B	Scale	05/04/2022
I2	1/2a	S3	A	Common	Operational FCS	Cutting	Zone B	Stainless steel guard	05/10/2021
I23	1/2a	S3	A	Deboned hams	Operational FCS	Deboning	Zone C	Membrane skinner	05/10/2021
I257	1/2a	S3	A	Deboned hams	Operational FCS	Deboning	Zone C	Plastic slat door	10/05/2022
I26	1/2a	S3	A	Deboned hams	Operational FCS	Deboning	Zone C	Plastic product storage boxes	05/10/2021
I337	1/2a	S3	A	Common	Operational FCS	Cutting	Zone B	Knife, glove, and knife sharpener set	25/10/2022
I342	1/2a	S3	A	Deboned hams	Operational FCS	Deboning	Zone C	Plastic slat door	25/10/2022
I4	1/2a	S3	A	Common	Operational FCS	Cutting	Zone B	Scale	05/10/2021
I61	1/2a	S3	A	Deboned hams	Final Product	Deboning	Zone C	Deboned fresh ham in pieces	03/11/2021
I8	1/2a	S3	A	Common	Operational FCS	Cutting	Zone B	Conveyor belt	05/10/2021
I81	1/2a	S3	A	Common	Operational FCS	Cutting	Zone B	Stainless steel rollers and table	09/11/2021
I86	1/2a	S3	A	Deboned hams	Operational FCS	Deboning	Zone C	Plastic slat door	09/11/2021
I108	1/2a	S4	A	Common	Operational FCS	Cutting	Zone B	Conveyor belt	25/01/2022
I111	1/2a	S4	A	Common	Operational FCS	Cutting	Zone B	Stainless steel rollers and table	25/01/2022
I17	1/2a	S4	A	Deboned hams	Operational FCS	Deboning	Zone C	Plastic slat door	05/10/2021
I196	1/2a	S4	A	Deboned hams	Operational FCS	Deboning	Zone C	Knife, glove, and knife sharpener set	05/04/2022
I29	1/2a	S4	A	Common	Pre-operational FCS	Cutting	Zone B	Conveyor belt	05/10/2021
I47	1/2a	S4	A	Deboned hams	Final Product	Deboning	Zone C	Deboned whole fresh ham	03/11/2021
I6	1/2a	S4	A	Common	Operational FCS	Cutting	Zone B	Conveyor belt	05/10/2021
P19	1/2a	S5	B	Burritos	Raw material	Pre-lethal	Zone B	Minced beef meat	26/10/2021
P28	1/2a	S5	B	Migas	Raw material	Pre-lethal	Zone A	Chorizo	16/11/2021
P30	1/2a	S5	B	Migas	Raw material	Pre-lethal	Zone A	Chorizo	16/11/2021
P60	1/2a	S5	B	Migas	Raw material	Pre-lethal	Zone A	Chorizo	22/03/2022
P97	1/2a	S5	B	Burritos	Raw material	Pre-lethal	Zone B	Minced beef meat	21/06/2022
I360	1/2a	S6	A	Common	NFCS	Cutting	Zone B	Drain	08/11/2022
I370	1/2a	S7	A	Common	NFCS	Storage	Zone D	Drain	08/11/2022
P101	1/2a	S8	B	Migas	Raw material	Pre-lethal	Zone A	Chorizo	28/06/2022
p69	1/2a	S8	B	Burritos	Raw material	Pre-lethal	Zone B	Minced beef meat	24/05/2022
P71	1/2a	S8	B	Burritos	Raw material	Pre-lethal	Zone B	Minced beef meat	24/05/2022
P74	1/2a	S8	B	Burritos	Raw material	Pre-lethal	Zone B	Minced beef meat	24/05/2022
P77	1/2a	S8	B	Burritos	Raw material	Pre-lethal	Zone B	Minced beef meat	24/05/2022
P80	1/2a	S8	B	Burritos	Raw material	Pre-lethal	Zone B	Minced beef meat	24/05/2022
P83	1/2a	S8	B	Migas	Raw material	Pre-lethal	Zone A	Chorizo	25/05/2022
P86	1/2a	S8	B	Migas	Raw material	Pre-lethal	Zone A	Chorizo	25/05/2022
P89	1/2c	S9	B	Burritos	Raw material	Pre-lethal	Zone B	Minced beef meat	07/06/2022
P134	1/2c	S9	B	Burritos	Operational FCS	Pre-lethal	Zone B	Beef mincer	14/2/2023
I112	1/2c	S9	A	Common	Operational FCS	Cutting	Zone B	Stainless steel rollers and table	25/01/2022
I334	1/2b	S10	A	Common	Operational FCS	Cutting	Zone B	Conveyor belt	25/10/2022
I340	1/2b	S11	A	Common	Operational FCS	Cutting	Zone B	Stainless steel rollers and table	25/10/2022
I351	1/2b	S11	A	Common	Pre-operational FCS	Cutting	Zone B	Stainless steel guard	25/10/2022
I76	1/2b	S11	A	Common	Operational FCS	Cutting	Zone B	Conveyor belt	09/11/2021
I367	1/2b	S11	A	Deboned hams	NFCS	Deboning	Zone C	Drain	08/11/2022
I19	1/2b	S12	A	Deboned hams	Operational FCS	Deboning	Zone C	Conveyor belt	05/10/2021
I95	4b	S13	A	Deboned hams	Operational FCS	Deboning	Zone C	Membrane skinner	09/11/2021
